# Parent-adolescent interaction and risk of adolescent internet addiction: a population-based study in Shanghai

**DOI:** 10.1186/1471-244X-14-112

**Published:** 2014-04-15

**Authors:** Jian Xu, Li-xiao Shen, Chong-huai Yan, Howard Hu, Fang Yang, Lu Wang, Sudha Rani Kotha, Fengxiu Ouyang, Li-na Zhang, Xiang-peng Liao, Jun Zhang, Jin-song Zhang, Xiao-ming Shen

**Affiliations:** 1 Xinhua Hospital, MOE-Shanghai Key Laboratory of Children’s Environmental Health, Department of Child and Adolescent Healthcare, Shanghai Institute for Pediatric Research, Shanghai Jiao Tong University School of Medicine, Shanghai, China; 2 Dalla Lana School of Public Health, University of Toronto, Toronto, Canada; 3 Global Health Department, University of Michigan, Ann Arbor, Michigan, USA; 4 Biostatistics Department, School of Public Health, University of Michigan, Ann Arbor, Michigan, USA; 5 Global Health at University of Toronto’s Faculty of Medicine, Toronto, Canada; 6 Biostatistics Department, Shanghai Jiao Tong University School of Medicine, Shanghai, China

**Keywords:** Adolescents, Internet addiction, Mother-child relations, Father-child relations, China, Marital status, Family structure

## Abstract

**Background:**

Family-based intervention is essential for adolescents with behavioral problems. However, limited data are available on the relationship between family-based factors and adolescent internet addiction (AIA). We aimed to examine this relationship using a representative sample of Shanghai adolescents.

**Methods:**

In October 2007, a total of 5122 adolescents were investigated from 16 high schools via stratified-random sampling in Shanghai. Self-reported and anonymous questionnaires were used to assess parent-adolescent interaction and family environments. AIA was assessed by DRM-52 Scale, developed from Young’s Internet-addiction Scale, using seven subscales to evaluate psychological symptoms of AIA.

**Results:**

Adjusting for adolescents’ ages, genders, socio-economic status, school performances and levels of the consumption expenditure, strong parental disapproval of internet-use was associated with AIA (*vs*. parental approval, *OR* = 2.20, 95% *CI*: 1.24-3.91). Worse mother-adolescent relationships were more significantly associated with AIA (*OR* = 3.79, 95% *CI*: 2.22-6.48) than worse father-adolescent relationships (*OR* = 1.76, 95% *CI*: 1.10-2.80). Marital status of “married-but-separated” and family structure of “left-behind adolescents” were associated with symptoms of some subscales. When having high monthly allowance, resident students tended to develop AIA but commuter students did not. Family social-economic status was not associated with the development of AIA.

**Conclusions:**

The quality of parent-adolescent relationship/communication was closely associated with the development of AIA, and maternal factors were more significantly associated with development of AIA than paternal factors. Family social-economic status moderated adolescent internet-use levels but not the development of AIA.

## Background

Rates of internet access were continuing to increase in both developing and developed countries [1-5]. With rapid rise in internet use over the past decade, and with adolescents’ immature physical and psychological development, adolescent internet addiction (AIA) is increasingly becoming a serious problem and has caused great concern from the public and specialists alike [1,2,6-10].

The conceptualization or definition of internet addiction was originally based on pathological gambling and substance use, but is still debated by academics and clinicians now [11]. Nonetheless, internet addiction is generally described in the psychological literature as an individual’s inability to control internet use, having the core symptoms including withdrawal reaction, preoccupation, tolerance, and significant functional impairments [11-14]. AIA reduced adolescent academic achievement, impaired psychological well-being of adolescents, and gave rise to psychosomatic symptoms and interpersonal problems in adolescents [8,15]. AIA may also induce structural changes in the brain regions including anterior cingulated cortex, orbitofrontal cortex and dorsolateral prefrontal cortex, or executive dysfunction such as elevated impulsivity and impaired cognitive control [13,16].

Identification of family characteristics of adolescent internet addicts is helpful for family-based intervention, considered the essential part of cognitive behavioral treatment and psychosocial support for adolescent internet addicts [11]. However, limited data are available on the relationship between AIA and family.

Poor family function was found to be one of the most important risk factors for addictive substance use and other severely abnormal adolescent behaviors [15,17-21]. Some studies have noted that family may play a role in the development of AIA [2,22], but many limitations existed in those studies. Firstly, maternal role and paternal role in the development of AIA were not separately discussed in detail before, and details on family economic levels were not questioned. Secondly, in previous studies, the independent risk factors for AIA from family environments were not explored after controlling for adolescent school performances and levels of the consumption expenditure. Finally, most of the previous studies were conducted in small and non-representative study populations, and the corresponding results may be biased due to non-probability sampling. Therefore, there is a need for more representative surveys [4,22,23].

The objective of this study was to identify the relationship between AIA and patterns of parent-adolescent interaction using a representative sample of Shanghai adolescents.

## Methods

### Study design and participants

The sample was recruited from junior and senior high schools in Shanghai from October to November 2007. The survey was an anonymous, self-administered and paper-and-pencil questionnaire, conducted in classroom settings.

Stratified and cluster random sampling was performed to ensure that participants were representative of the overall adolescent population in Shanghai. Sampling method was described in detail before [5]. We randomly selected 6 administrative districts out of 19 administrative districts in Shanghai depending on their geographical features, socioeconomic characteristics, and population density. Within the 6 districts, in urban or suburban area, we randomly selected 2 junior high schools and 6 senior high schools (2 ordinary, 2 key, and 2 vocational). All the students in 7^th^-12^th^ grades in each school were enrolled. Therefore, in total, 16 high schools and 5135 adolescents were recruited. Thirteen subjects were excluded based on missing data on more than 3 variables in general information questionnaire or due to missing data in DRM-52 Scale. This resulted in a total sample of 5122 adolescents (response rate: 99.8%).

School approval and parental informed consent (written) were obtained at the study’s initiation. The study received approval from the medical ethical committee of Xinhua Hospital affiliated to Shanghai Jiao Tong University School of Medicine.

### Variables

The questionnaire contained 3 parts: 1) individual adolescent information included age, gender, grade, district and the type of school, 2) family factors, self-assessed levels of academic achievement and levels of monthly consumption expenditure that may potentially affect internet use, 3) adolescent behaviors of internet usage. Familial background variables included parental education levels, occupations, marital status, family structure, residence rented or owned, having cars/computers/internet access at home or not, adolescent having private bedrooms or not, only child or not, residential or commuter students, adolescents’ monthly allowance levels. Quality of the parent-adolescent relationship was evaluated with data from answers to the following questions: What is your perception of the quality of your mother-father relationship? Of your relationship with your mother? Of your relationship with your father? What is your perception of parental attitude toward your internet use?

### Measures

AIA was assessed by DRM-52 Scale, developed from Young’s Internet-Addiction Scale and adapted for use in Shanghai [5,12,24]*.* The scale included all contents of Young’s scale, used both direct and indirect questions to collect data, and used seven subscales to evaluate psychological symptoms of AIA. A detailed description of the contents of seven subscales and psychometric properties of the scale was described before [5,24].

The total score on DRM-52 Scale ranged from 0 to 260. A score of zero meant that an adolescent never used the internet, a score over 163 was defined as internet addiction, and higher scores indicated increasing severity of internet addiction [5,24].

### Statistical analysis

For univariate analyses, a one-way analysis of variance (ANOVA) was used to analyze the differences of total scores of DRM-52 Scale and Chi-square was used to compare the prevalence rates of AIA or internet-use among different levels of the same family background variable. For multivariable regression, binary logistic regression and multiple linear regression analyses (stepwise model) were both adopted to evaluate the strength of association between family factors and AIA, and linear regression was also used to evaluate the association between family factors and symptoms of seven subscales. In the first stage, Base Model 1 included all family background variables mentioned above (in “*Variables*”); Base Model 2 included adolescent personal variables such as adolescents’ genders, grades, types of schools, levels of academic achievement and levels of monthly consumption expenditure [5]. To control for potential confounding, we used another two models to evaluate the effects of family on the development of AIA. Model 1 adjusted for all the personal variables included in Base model 2 which were shown to be independent risk factors for AIA [5]. Because possible relationship between family structure, marital status, and AIA were mentioned in previous studies [2,18,21,22], Model 2 was performed to force two additional variables including family structure and marital status into the model. Data stratification by adolescent boarding status and monthly allowance was performed. The statistical significance was set at *p* <0.05 (two tailed).

## Results

Of the 5122 participants, the mean age of respondents was 15.9 years with a range from 11.3 to 20.4 years, and the boy/girl ratio was close to 1:1 (2542 boys, 49.6%; 2580 girls, 50.4%). As shown in Tables 1 and 2, the sample was from diverse socioeconomic backgrounds.

**Table 1:** Family characteristics of the study population of 5122 adolescents in Shanghai

**Variables**	**N (%)**	**Prevalence of Internet use (%)**^**a**^	**Total scores**^**b**^	**Prevalence of AIA (%)**^**c,d**^
**Paternal education (missing 23)**		*p* <0.001	*p* <0.001	*p =*0.01
Illiteracy and elementary	101 (2.0%)	84.2% (85/101)	112.3 ± 53.7	9.9% (10/101)
Junior high school	1115 (21.8%)	92.8% (1035/1115)	116.9 ± 43.3	8.7% (97/1115)
Senior high school	2362 (46.1%)	94.9% (2242/2362)	122.8 ± 39.1	10.0% (235/2362)
University-level and beyond	1521 (29.7%)	93.6% (1424/1521)	117.4 ± 40.5	6.8% (104/1521)
**Maternal education (missing 26)**		*p =*0.002	*p <*0.001	*p =*0.001
Illiteracy and elementary	147 (2.9%)	91.2% (134/147)	110.8 ± 44.0	4.1% (6/147)
Junior high school	1215 (23.7%)	92.2% (1120/1215)	116.4 ± 43.6	8.7% (106/1215)
Senior high school	2327 (45.4%)	94.8% (2206/2327)	123.2 ± 39.5	10.3% (239/2327)
University-level and beyond	1407 (27.5%)	94.0% (1323/1407)	117.6 ± 40.0	6.9% (97/1407)
**Family structure (missing 0)**		*p* =0.22	*p* =0.01	*p* =0.01
Nuclear family	3380 (66.0%)	93.5% (3158/3380)	118.9 ± 41.2	8.5% (288/3380)
Three-generation family	1069 (20.9%)	95.0% (1016/1069)	118.9 ± 38.9	7.5% (80/1069)
Single parent family	357 (7.0%)	93.6% (334/357)	124.3 ± 44.0	13.2% (47/357)
Left-behind adolescents	181 (3.5%)	93.9% (170/181)	124.7 ± 43.5	12.2% (22/181)
Weekend parents	135 (2.6%)	96.3% (130/135)	126.8 ± 35.1	9.6% (13/135)
**Parental marriage (missing 4)**		*p* =0.23	*p* =0.003	*p* =0.004
Married & together	4477 (87.4%)	93.9% (4202/4477)	119.0 ± 40.5	8.3% (370/4477)
Married-but-separated	81 (1.6%)	93.8% (76/81)	132.5 ± 44.2	17.3% (14/81)
Divorced	360 (7.0%)	93.6% (337/360)	124.2 ± 43.5	12.2% (44/360)
Widowed	86 (1.7%)	90.7% (78/86)	117.5 ± 47.1	10.5% (9/86)
Remarried	114 (2.2%)	97.4% (111/114)	125.6 ± 35.9	11.4% (13/114)
**Commuter students or not (missing 13)**		*p* =0.03	*p* =0.002	*p* =0.67
Resident students	451 (8.8%)	96.0% (433/451)	125.2 ± 35.6	9.5% (43/451)
Commuter students	4668 (91.2%)	93.7% (4372/4668)	119.2 ± 41.3	8.7% (407/4668)
**Only child (missing 12)**		*p =*0.34	*p =*0.21	*p =*0.56
Yes	4658 (90.9%)	94.0% (4377/4658)	119.9 ± 40.8	8.9% (413/4658)
No	452 (8.8%)	92.9% (420/452)	117.4 ± 42.0	8.0% (36/452)
**Family housing (missing 18)**		*p* <0.001	*p* =0.01	*p* =0.27
Own	4724 (92.2%)	94.3% (4455/4724)	120.1 ± 40.3	9.0% (423/4724)
Rent	380 (7.4%)	89.0% (338/380)	114.4 ± 46.9	6.8% (26/380)
**Having computers at home (missing 3)**		*p* <0.001	*p* <0.001	*p* <0.001
Yes	4359 (85.1%)	96.5% (4208/4359)	123.9 ± 36.4	9.7% (422/4359)
No	760 (14.8%)	78.6% (597/760)	95.2 ± 54.4	3.7% (28/760)
**Having private bedroom (missing 9)**		*p* <0.001	*p =*0.02	*p =*0.32
Yes	4169 (81.4%)	94.5% (3940/4169)	120.3 ± 39.7	8.6% (360/4169)
No	944 (18.4%)	91.1% (860/944)	116.8 ± 45.6	9.3% (88/944)

^a^Prevalence of Internet use: the ratio of the number of adolescents using internet to the number of the whole adolescent sample in that group. Chi-square was used to compare the prevalences of internet-use among different levels of the same family background variable.

^b^Total scores: total scores of DRM-52 Scale. ANOVA was used to analyze the differences of total scores of DRM-52 Scale among different levels of the same family background variable.

^c^AIA = adolescent internet addiction.

^d^Prevalence of AIA: the ratio of the number of internet-addicted adolescents to the number of the whole adolescent sample in that group. Chi-square was used to compare the prevalences of AIA among different levels of the same family background variable.

**Table 2:** **Parent-adolescent interaction and AIA in 5122 Shanghai adolescents**^**a**^

**Variables**	**N (%)**	**Prevalence of internet use (%)**^**b**^	**Total scores**^**c**^	**Prevalence of AIA (%)**^**d**^
**Father-mother relationship (missing 27)**		*p =*0.75	*p* <0.001	*p* <0.001
Very good	3640 (71.1%)	93.7% (3410/3640)	116.5 ± 40.4	6.7% (243/3640)
Relatively good	794 (15.5%)	94.1% (747/794)	127.8 ± 41.0	14.1% (112/794)
General	379 (7.4%)	94.2% (357/379)	127.6 ± 42.4	15.6% (59/379)
Relatively & very bad	282 (5.5%)	95.0% (268/282)	127.3 ± 41.0	12.4% (35/282)
**Father-adolescent relationship (missing 19)**		*p* =0.87	*p* <0.001	*p* <0.001
Very good	2691 (52.5%)	93.7% (2521/2691)	114.5 ± 40.3	6.1% (163/2691)
Relatively good	1332 (26.0%)	93.9% (1251/1332)	122.7 ± 39.9	10.2% (136/1332)
General	888 (17.3%)	94.4% (838/888)	128.2 ± 40.8	12.6% (112/888)
Relatively & very bad	192 (3.8%)	93.2% (179/192)	130.0 ± 45.1	18.2% (35/192)
**Mother-adolescent relationship (missing 17)**		*p* =0.71	*p* <0.001	*p* <0.001
Very good	3137 (61.3%)	94.1% (2953/3137)	115.4 ± 39.7	6.28% (197/3137)
Relatively good	1353 (26.4%)	93.4% (1263/1353)	125.0 ± 41.7	11.5% (155/1353)
General	522 (10.2%)	93.1% (486/522)	127.8 ± 42.2	12.6% (66/522)
Relatively & very bad	93 (1.8%)	95.7% (89/93)	137.3 ± 44.1	31.12% (29/93)
**Parental attitude toward internet use (missing 18)**		*p* <0.001	*p* =0.004	*p* <0.001
Agree	441 (8.6%)	95.5% (421/441)	119.7 ± 38.4	7.0% (31/441)
Relatively agree	1688 (33.0%)	96.9% (1635/1688)	119.4 ± 33.6	6.3% (107/1688)
General	1437 (28.1%)	93.7% (1346/1437)	120.2 ± 41.2	8.6% (124/1437)
Relatively disagree	1312 (25.6%)	92.2% (1209/1312)	121.7 ± 44.7	11.5% (151/1312)
Strongly disagree	226 (4.4%)	82.7% (187/226)	110.3 ± 59.2	15.5% (35/226)
**Adolescent monthly allowance (missing 14)**		*p* <0.001	*p* <0.001	*p* <0.001
<100 RMB	2776 (54.2%)	92.2% (2561/2776)	112.0 ± 42.3	6.5% (179/2776)
100 ~ 299 RMB	1572 (30.7%)	95.5% (1503/1572)	127.0 ± 37.6	10.6% (167/1572)
300 ~ 599 RMB	541 (10.6%)	97.8% (529/541)	132.0 ± 33.3	12.9% (70/541)
>600 RMB	219 (4.3%)	93.6% (205/219)	132.8 ± 42.6	15.1% (33/219)

^a^AIA = adolescent internet addiction.

^b^Prevalence of Internet use: the ratio of the number of adolescents using internet to the number of the whole adolescent sample in that group. Chi-square was used to compare the prevalences of internet-use among different levels of the same family background variable.

^c^Total scores: total scores of DRM-52 Scale. ANOVA was used to analyze the differences of total scores of DRM-52 Scale among different levels of the same family background variable.

^d^Prevalence of AIA: the ratio of the number of internet-addicted adolescents to the number of the whole adolescent sample in that group. Chi-square was used to compare the prevalences of AIA among different levels of the same family background variable.

### Bivariate associations between family background variables and adolescent internet-use/internet-addiction

Adolescent internet-use levels were found to be associated with factors relating to family’s social-economic status (SES). Adolescents from high SES families had higher internet-use levels than adolescents from low SES families (Table 1).

Of parent-adolescent interaction patterns, compared with adolescents whose parents approved of their internet use, adolescents who perceived strong parental disapproval had lower internet-use levels but with higher total scores and a higher prevalence of AIA. Adolescents with worse relationships with the mother or with the father had higher total scores and a higher prevalence of AIA. Of marital status, adolescents from married-but-separated families had the highest total scores and the highest prevalence of AIA (Table 2).

### Adjusted associations between family background variables and adolescent internet-use/internet-addiction

After controlling for confounding influences, both multivariate linear regression and logistic regression analyses showed that adolescents whose parents strongly disapproved of their internet-use had higher risks of AIA than adolescents whose parents approved (odds ratio (*OR*): 2.20, 95% confidential interval (*CI*): 1.24-3.91, Table 3), and were significantly associated with symptoms of 6 subscales (however, not significantly associated with the Time-Consuming symptom) (Table 4). When compared to adolescents who had good mother relationships, adolescents with bad mother relationships were more likely to develop AIA (*OR* 3.79, 95% *CI*: 2.22-6.48, Table 3), and had corresponding symptoms in Socialization, Planning, Negative-Life-Consequences and Tolerance subscales (Table 4). Bad father-adolescent relationship was associated with AIA (*OR* 1.76, 95% *CI*: 1.10-2.80) and with the symptom of Withdrawal subscale (Tables 3 and 4).

**Table 3:** **Logistic regression analysis results for the association between parent-adolescent interaction and AIA development**^**a,b**^

**Potential risk factors**	**N**	**Odds ratio (95% CI)**	
		**Base Model 1**^**c **^**(Un-adjusted)**	**Model 2**^**d **^**(Adjusted)**
**Parental attitude toward adolescent internet use**	5104		
Agree	441	1.0 (Reference)	1.0 (Reference)
Relatively agree	1688	0.90(0.59-1.36)	1.05(0.67-1.64)
General	1437	1.25(0.83-1.88)	1.08(0.69-1.69)
Relatively disagree	1312	1.72(1.15-2.57)**^e^	1.54(0.98-2.40)
Strongly disagree	226	2.42(1.45-4.05)**^e^	2.20(1.24-3.91)**^e^
**Mother-adolescent relationship**	5105		
Very good	3137	1.0 (Reference)	1.0 (Reference)
Relatively good	1353	1.93(1.55-2.41)***	1.25 (0.95-1.66)
General	522	2.16(1.61-2.90)***	1.20 (0.83-1.73)
Very & relatively bad	93	6.76(4.26-10.73)***	3.79 (2.22-6.48)***
**Father-adolescent relationship**	5103		
Very good	2691	1.0 (Reference)	1.0 (Reference)
Relatively good	1332	1.76(1.39-2.24)***	1.32(0.98-1.76)
General	888	2.24(1.74-2.89)***	1.39(0.99-1.93)
Very & relatively bad	192	3.46(2.32-5.15)***	1.76(1.10-2.80)*
**Parental marriage**	5118		
Married-and-together	4477	1.0 (Reference)	1.0 (Reference)
Married-but-separated	81	2.32(1.29-4.17)**	2.06(1.05-4.06)*
Divorced	360	1.55(1.11-2.16)*	1.14 (0.74-1.75)
Widowed	86	1.30(0.65-2.61)	1.52 (0.66-3.47)
Remarried	114	1.43(0.79-2.57)	1.13 (0.61-2.10)
**Family structure**	5122		
Nuclear family	3380	1.0 (Reference)	1.0 (Reference)
Three-generation family	1069	0.87(0.67-1.12)	0.84(0.64-1.11)
Single-parent family	357	1.63(1.17-2.26)*	1.19(0.79-1.79)
Left-behind adolescents	181	1.49(0.94-2.37)	1.26(0.76-2.08)
Weekend parents	135	1.14(0.64-2.05)	0.94(0.50-1.76)
**Monthly allowance levels**	5108		
**Among resident students**	451		
<100 RMB/month	105	1.0 (Reference)	1.0 (Reference)
100 ~ 299 RMB/month	154	0.66(0.24-1.83)	0.59(0.20-1.76)
300 ~ 599 RMB/month	131	1.34(0.53-3.36)*	1.15(0.43-3.13)
>600 RMB/month	61	3.61(1.42-9.21)**	3.60(1.27-10.20)*
**Among commuter students**	4657		
<100 RMB/month	2671	1.0 (Reference)	1.0 (Reference)
100 ~ 299 RMB/month	1418	1.85(1.47-2.32)***	1.64(1.28-2.11)***
300 ~ 599 RMB/month	410	2.36(1.72-3.25)***	1.75(1.22-2.50)**
>600 RMB/month	158	2.00(1.21-3.31)**	1.32(0.76-2.31)

^a^AIA = adolescent internet addiction.

^b^The logistic regressions were fitted to model the possibility of adolescent having AIA. We set ‶0″ for adolescents with total scores of DRM 52 scale <163 and ‶1″ for adolescents with total scores ≥163.

^c^In Base Model 1, only family background variables were included.

^d^In model 2, family background variables (same variables in Base Model 1) and other related variables including grades, types of schools, monthly consumption expenditure levels, academic achievement levels and family social economic status were adjusted. Two variables including family structure and marital status were forced in this model.

^e^***indicated *p* < 0.001, **indicated *p* < 0.01, *indicated *p* < 0.05.

**Table 4:** **Linear regression results for the associations between family factors and development of AIA/AIA symptoms**^**a,b**^

**Potential risk factors**	**Total score**^**c**^	**Seven subscale scores**^**c**^						
		**Lack of Control**	**Socialization**	**Planning**	**Negative-Life-Consequences**	**Time-Consuming**	**Tolerance**	**Withdrawal**
**Parental attitude toward adolescent internet use**								
Agree	Reference	Reference	Reference	Reference	Reference	Reference	Reference	Reference
Relatively agree	1.0(1.5)^d^	0.5(0.2)^d^	0.6(0.3)^d^	0.2(0.2) ^d^	0.1(0.3)^d^	-0.2(0.2)^d^	-0.0(0.2)^d^	-0.2(0.5)^d^
General	0.7(1.5)	0.4(0.2)	0.6(0.3)	0.4(0.2)	0.4(0.3)	-0.2(0.2)	-0.3(0.2)	-0.6(0.6)
Relatively disagree	5.8(1.6)***^e^	1.1(0.2)***^e^	1.8(0.3)***^e^	1.1(0.2)*** ^e^	1.1(0.3)***^e^	-0.1(0.2)	0.4(0.2)*^e^	0.5(0.6)
Strongly disagree	11.1(2.5)***	1.4(0.3)***	2.4(0.5)***	1.7(0.4)***	1.7(0.4)***	-0.3(0.4)	1.0(0.3)***	2.2(0.9)*
**Mother-adolescent relationship**								
Very good	Reference	Reference	Reference	Reference	Reference	Reference	Reference	Reference
Relatively good	5.3(0.9)***	0.4(0.2)	1.0(0.2)***	0.9(0.1)***	1.0(0.2)***	0.1(0.2)	0.6(0.1)***	0.7(0.4)
General	5.2(1.4)***	-0.1(0.2)	1.1(0.3)***	0.6(0.2)**	1.0(0.2)***	0.1(0.2)	0.6(0.2)***	1.0(0.6)
Relatively & very bad	12.0 (3.2)***	0.7(0.5)	2.4(0.7)***	1.4(0.5)**	1.5(0.6)**	1.0(0.5)	1.3(0.4)**	2.0(1.2)
**Father-adolescent relationship**								
Very good	Reference	Reference	Reference	Reference	Reference	Reference	Reference	Reference
Relatively good	2.6(1.1)	0.0(0.2)	0.5(0.2)	0.3(0.2)	0.4(0.2)	0.0(0.3)	0.5(0.3)	1.3(0.3)***
General	4.7(2.3)	0.3(0.2)	0.6(0.3)	0.5(0.2)	0.4(0.2)	0.2(0.2)	0.1(0.2)	2.2(0.4)***
Relatively & very bad	4.2(2.2)	0.1(0.3)	0.8(0.5)	0.4(0.3)	0.5(0.4)	0.2(0.1)	-0.3(0.2)	2.8(0.8)***
**Parental marriage**								
Married-and-together	Reference	Reference	Reference	Reference	Reference	Reference	Reference	Reference
Married-but-separated	8.4(3.4)*	1.1(0.5)*	1.3(0.7)	0.8(0.5)	0.5(0.6)	0.1(0.5)	0.8(0.4)	3.5(1.2)**
Divorced	0.9(2.4)	0.3(0.3)	0.0(0.5)	0.0(0.3)	-0.2(0.4)	0.3(0.3)	0.1(0.3)	0.1(0.9)
Widowed	1.4(4.3)	0.1(0.6)	0.1(0.9)	0.4(0.6)	-0.3(0.8)	0.5(0.6)	-0.1(0.5)	0.7(1.6)
Remarried	-0.0(2.3)	0.2(0.4)	-0.2(0.5)	0.2(0.4)	-0.3(0.5)	0.2(0.4)	-0.5(0.3)	0.9(1.0)
**Family structure**								
Nuclear family	Reference	Reference	Reference	Reference	Reference	Reference	Reference	Reference
Three-generation family	-1.7(1.0)	-0.2(0.1)	-0.3(0.2)	-0.2(0.1)	-0.3(0.2)*	-0.0(0.1)	-0.3(0.1)*	-0.4(0.4)
Single parent family	0.8(2.6)	-0.1(0.4)	0.2(0.5)	0.0(0.4)	0.4(0.5)	0.1(0.4)	0.2(0.3)	-0.1(1.0)
Left-behind adolescents	-0.3(2.3)	-0.1(0.3)	-0.5(0.5)	-0.1(0.3)	0.3(0.4)	0.9(0.3)**	-0.3(0.3)	-0.7(0.8)
Weekend parents	1.9(2.4)	0.5(0.3)	0.7(0.5)	0.1(0.4)	-0.4(0.4)	0.0(0.4)	0.1(0.3)	1.0(0.9)

^a^AIA = adolescent internet addiction.

^b^Linear regressions were used to model the relationship between family factors and AIA and between family factors and symptoms of 7 subscales. Total scores and subscale scores of DRM-52 Scale were respectively taken as dependent variables. Adjusted R squares for these models were around 0.3.

^c^In these models, adolescent gender, age, grade, the type of school, monthly consumption expenditure, academic achievement levels and family social economic status were adjusted. Two variables including family structure and marital status were forced.

^d^Results are reported as Coefficient Estimate (SE).

^e^***indicated *p* < 0.001, **indicated *p* < 0.01, *indicated *p* < 0.05.

In Model 2, we found significant association between marital status of married-but-separated and AIA (*OR*: 2.06, 95% *CI*: 1.05-4.06, Table 3), and between marital status of married-but-separated and significant symptoms in Lack-of-Control and Withdrawal subscales (Table 4), and association between family structure of three-generation families and fewer symptoms in Negative-Life-Consequence and Tolerance subscales, as well as significant association between family structure of left-behind adolescents and Time-Consuming subscale (Table 4). Results indicated a marginal association between the total scores of DRM-52 Scale and variables of family structure and marital status, suggesting that these two variables were potential but not strong risk factors for AIA.

For resident students, high monthly allowance was associated with higher risk of AIA (> 600 RMB/month *vs* < 100 RMB/month, *OR*: 3.60, 95% *CI*: 1.27-10.20). However, for commuter students, adolescents with high monthly allowance were not significantly associated with AIA (Table 3).

No factors related to family SES entered any final regression models.

## Discussion

This study demonstrated that parent-adolescent interactions played an important role in the development of AIA. However, familial SES had no significant impact on the development of AIA.

### Parent-adolescent interaction compared to family SES in the development of AIA

Our study showed that strong parental disapproval of adolescent internet use was significantly associated with the development of AIA. This may be explained by the poor quality of parent-adolescent communication such that adolescents were unwilling to take advice from their parents. Previous studies [25] found that adolescents’ perceptions of parental disapproval of their engaging in sex might be associated with increased risk for adolescent sexual behavior, and adolescent perceptions of parental attitudes tended to be more predictive of risky sexual behavior than actual parental attitudes. This trend was verified in our study that showed the importance of improving parent-adolescent communication.

Few studies discussed the role of family economic status in AIA. The result of one small study was consistent with ours in that family SES had no significant effects on AIA [22]. As for the role of monetary allowance in adolescent behaviors, one previous paper reported significant association between high monthly allowance and smoking in young urban Malaysian women [26]. In this study, high monthly allowance was a risk factor for AIA in resident students, but not a risk factor for AIA in commuter students. Due to relative freedom from parental supervision, high monthly allowance may provide opportunities to resident students to frequently use internet. “Peer effects” (peer influences on drinking, smoking, and frequent internet usage) may also have significant impacts on development of AIA in resident students [27]. However, for commuter students, their parents usually had more opportunities to supervise their spending of money, in general, their daily living expenses had been paid by their parents directly. Therefore, higher monthly allowance might not be related to more monthly spending in commuter students, rather, it reflect the common parental confidence in those adolescents. This result also suggested the importance of parental supervision and parent-adolescent harmonious interaction in prevention of AIA.

### The maternal and paternal roles in guiding adolescent internet use

Maternal factors were found to play a central role in guiding adolescents in appropriate internet use. A previous study contrasting 72 mother-child dyads in the Netherlands and another study investigating 635 Arab Muslim immigrant mother-adolescent pairs both found that mother-adolescent relationship affected adolescent behavior adjustment greatly [28,29]. Another study of 55 Latino families revealed that mother-adolescent communication affected both adolescent behaviors and attitudes toward premarital sex and adolescents’ perceptions of openness in the mother-adolescent relationship [30]. These studies were consistent with our results in showing the importance of improving mother-adolescent relationship in adjusting adolescent behaviors.

In our study, poor father-adolescent relationships were associated with the development of AIA, but were less significantly associated with AIA than maternal factors. This might suggest that, when compared with mothers, fathers in Chinese culture remain underrepresented in most child-related activities.

### Effects of marital status and family structure on AIA

Our study showed that adolescents from three-generation families were less likely to develop symptoms of AIA than those from other kinds of family structures. This was consistent with previous studies [17,19] and appeared to be due to supervision from both parents and grandparents.

Our study also showed that parental marital status of married-but-separated was marginally associated with AIA. One possible explanation is that adolescents might suffer from conflict or neglect as the separate status was maintained, and related emotional or behavioral problems might occur. Internet addiction might therefore develop due to the lack of parental supervision and adolescent emotional problems, similar to findings in previous studies [2,11,18,21,22,25].

Our study also showed that left-behind adolescents were prone to developing symptoms of AIA. Over the past twenty years, China has experienced rapid urbanization as more and more parents migrated from rural areas to cities or went abroad to enhance their careers or improve their economic condition. As a result, more adolescents were left behind. The prevalence of internet addiction among left-behind adolescents was most probably due to lack of parental care and supervision [31,32].

### Limitations

Firstly, there is no consensus on the diagnostic criteria for internet addiction. Although DRM-52 Scale was meant to absorb the essence of former instruments [11,12,33-35] and had relatively satisfactory psychometric properties, this scale might not be totally comprehensive and might need further refinement. The second limitation may be our reliance on data reported by adolescents themselves. Although anonymous questionnaires should have guaranteed confidentiality, a reporting bias is still possible. Thirdly, adolescents’ perception of parent-adolescent relationship might not fully reflect the real situation [29]. Fourthly, we may miss some other family factors relating to AIA that were not included as variable candidates in our questionnaire. Finally, our cross-sectional study raised the possibility of reverse causality, for example, that AIA may be a risk factor for worse mother-adolescent relationships (rather than the reverse).

## Conclusion

To the best of our knowledge, this is the first detailed report on the relationship between adolescent internet use or addiction and patterns of parent-adolescent interaction. Our findings demonstrated that the quality of parent-adolescent (especially mother-adolescent) relationship/communication is closely associated with the development of AIA, but family SES or family structure is not. The present findings may be applied to family-based prevention and early intervention of AIA.

## Competing interests

All authors have no competing interests to declare.

## Authors’ contributions

JX, CHY and XMS designed the study and supervised the data collection; JX drafted the manuscript; JX, LXS, XPL and JSZ undertook data collection; HH, SRK, FY, LW and LNZ conducted the statistical analyses; JX, LXS, CHY, HH, FXO, JZ and XMS assisted with the interpretation of results and revision for intellectual content. All authors read and approved the final manuscript.

## Pre-publication history

The pre-publication history for this paper can be accessed here:

http://www.biomedcentral.com/1471-244X/14/112/prepub
